# GWAS Hits for Bilateral Convergent Strabismus with Exophthalmos in Holstein Cattle Using Imputed Sequence Level Genotypes

**DOI:** 10.3390/genes12071039

**Published:** 2021-07-04

**Authors:** Anke Bögeholz, Clemens Falker-Gieske, Monika Guélat, Corinne Gurtner, Sibylle Hunziker, Anna Oevermann, Georg Thaller, Cord Drögemüller, Jens Tetens

**Affiliations:** 1 Department of Animal Sciences, Georg-August-University Göttingen, Burckhardtweg 2, 37077 Göttingen, Germany; anke.boegeholz@agr.uni-goettingen.de (A.B.); clemens.falker-gieske@uni-goettingen.de (C.F.-G.); 2 Clinic for Ruminants, Vetsuisse Faculty, University of Bern, Bremgartenstr. 109a, 3012 Bern, Switzerland; monika.guelat@bluewin.ch; 3 Institute of Animal Pathology, Vetsuisse Faculty, University of Bern, Länggassstr. 124, 3012 Bern, Switzerland; corinne.gurtner@vetsuisse.unibe.ch; 4 Institute of Genetics, Vetsuisse Faculty, University of Bern, Bremgartenstr. 109a, 3012 Bern, Switzerland; sc_hunziker@hotmail.com (S.H.); cord.droegemueller@vetsuisse.unibe.ch (C.D.); 5 Division of Neurological Sciences, Vetsuisse Faculty, University of Bern, Bremgartenstr. 109a, 3012 Bern, Switzerland; anna.oevermann@vetsuisse.unibe.ch; 6 Institute of Animal Breeding and Husbandry, Christian-Albrechts-University, Hermann-Rodewald-Str. 6, 24118 Kiel, Germany; gthaller@tierzucht.uni-kiel.de; 7 Center for Integrated Breeding Research, Georg-August-University Göttingen, Albrecht-Thaer-Weg 3, 37075 Göttingen, Germany

**Keywords:** bos taurus, eye disorder, development, rare disease, monogenic inheritance, histopathology, GWAS, imputation

## Abstract

Bilateral convergent strabismus with exophthalmos (BCSE) is a malformation of the eyes and is recognized as a mild but progressive disorder that affects cattle in the first two years of life. This most likely inherited disorder is rarely described in cattle resembling autosomal dominantly inherited forms of human progressive external ophthalmoplegia (PEO). In German Braunvieh cattle, two linked genome regions were found that could be responsible for the development and/or progression of BCSE. The goal of this study was to phenotypically characterize BCSE in Holstein cattle from Germany and Switzerland as well as to identify associated genome regions by GWAS. The clinicopathological phenotype of 52 BCSE-affected Holstein cattle was in accordance with the phenotype described in German Braunvieh cattle, but in addition, signs of degeneration and cellular infiltration in the eye muscles were found. By using imputed sequence level genotype data, three genome-wide significant GWAS hits were revealed on different chromosomes that were not detected by initial GWAS based on high density SNP array data highlighting the usefulness of this approach for mapping studies. The associated genome regions include the *ABCC4* gene as well as markers adjacent to the *NCOR2* and *DNAJC3* genes all illustrating possible functional candidate genes. Our results challenge a monogenic mode of inheritance and indicate a more complex inheritance of BCSE in Holstein cattle. Furthermore, in comparison to previous results from German Braunvieh cattle, it illustrates an obvious genetic heterogeneity causing BSCE in cattle. Subsequent whole genome sequencing (WGS)-based analyses might elucidate pathogenic variants in the future.

## 1. Introduction

**Figure 1:**
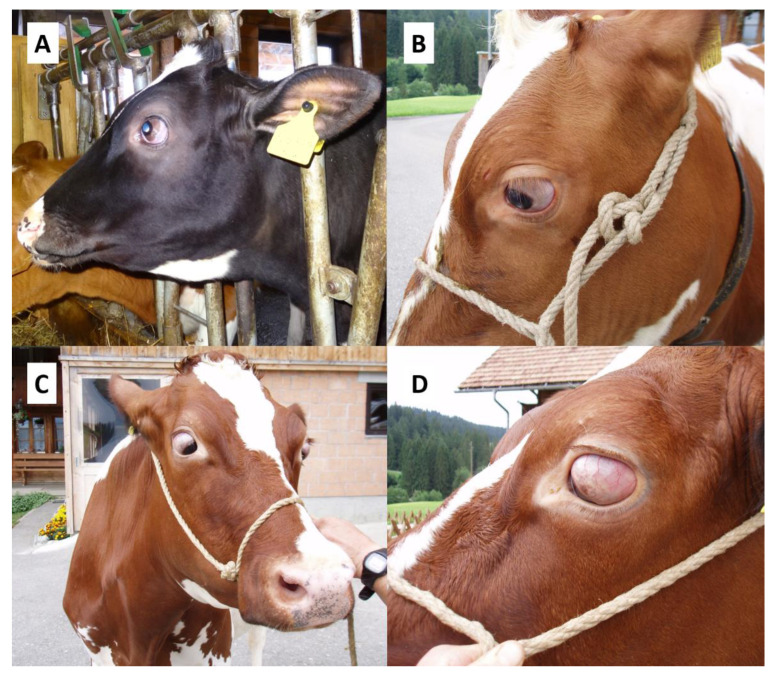
Examples of BCSE-affected Holstein cattle. (**A**) Nine-month old female Holstein heifer with moderate BCSE; (**B**). Eight-month old female Red Holstein heifer with moderate BCSE; (**C**,**D**) Two-and-a-half year old Red Holstein cow in first lactation with severe BCSE. The first two cases were subjected to histopathological and neuropathological examination.

Bilateral convergent strabismus with exophthalmos (BCSE) is a most likely hereditary eye defect in cattle, which has been described in different breeds like Jersey, German Braunvieh, German Fleckvieh and Holstein cattle affecting animals at different ages [1,2,3,4]. Affected animals exhibit a progressive mostly bilateral symmetric anterio-medial rotation of the eyes associated with a variable protrusion of the eyeballs [3,5]. The fixation of the bulbi causes visual impairment [1,3] and can lead to complete blindness in advanced cases due to the disappearance of the pupils behind the anterio-medial angle of the orbit [6] (Figure 1). A significantly decreased number of neurons in the core region of the *N. abducens* (VIth cranial nerve) that controls the movement of the *M. rectus lateralis* was observed that leads to motoric insufficiency of the innervated muscles [3]. Histopathological examinations of the *Mm. recti lateralis et medialis* later revealed the presence of so called “ragged-red-fibers” (RRF), a pathological accumulation of degenerated mitochondria in affected fibers, which indicates a lack of energy supply [7].

The age of onset of bovine BCSE varies, but in most cases, clinical findings were not reported until the age of six months. Frequently, early symptoms do not occur until the age of one to two years or even later in life [1,2,3,6,8]. The impaired vision in affected animals leads to changes in behavior such as unsecure gait, jumpiness and shyness [3,9]. This causes problems in handling especially in loose housing systems or when animals are turned out to pasture from tie-stalls. Therefore, otherwise healthy animals have to be culled early. As the disorder usually manifests in adult animals, rearing costs have been spent and potential carriers have already been used for breeding.

A genetic origin for bovine BCSE is suspected although the exact mode of inheritance as well as the molecular cause of BCSE remain unknown. Segregation analyses performed in German Braunvieh cattle showed that a model of a single dominantly inherited autosomal gene could best explain the observed segregation of BCSE [8]. In a microsatellite-based genome scan in Brown Swiss cattle, two BCSE-linked genome regions were identified on bovine chromosomes 5 and 18, respectively [5]. Based on these findings, two positional candidate genes were identified to be associated with BCSE, but so far no causative variant was found [10]. In addition, three genes implicated in different forms of autosomal dominant progressive external ophthalmoplegia (PEO; OMIM 157640), a very similar condition in humans with progressive bilateral ptosis and diffuse symmetric reduction in ocular motility [11], were analyzed as functional candidate genes for the occurrence of BCSE in German Braunvieh cattle, but no associated variants were identified [12].

Considering that previous genetic studies were limited to German Braunvieh cattle, the aim of the current study was to phenotypically characterize BCSE-affected Holstein cattle as well as to identify genome regions associated with BCSE in this breed. A GWAS approach based on high density SNP array data as well as imputed sequence level genotypes was applied.

## 2. Materials and Methods

### 2.1. Animals and Clinical Phenotypes

A total of 73 German and Swiss Holstein cows from different herds sporadically reported with eye anomalies resembling BCSE, but otherwise healthy, were examined and EDTA blood samples for DNA extraction were drawn. A total of 52 clinically unambiguously affected animals were selected as cases for the genetic study, based on the following three criteria: (I) bilateral manifestation, (II) simultaneous occurrence of convergent strabismus and exophthalmos and (III) an age of onset of more than 6 months. Deep pedigree data of the cases were partly available. In addition, 95 control animals of more than two years of age with no reported history of BCSE were selected from our data repository.

### 2.2. Histo- and Neuropathological Examinations

**Figure 2:**
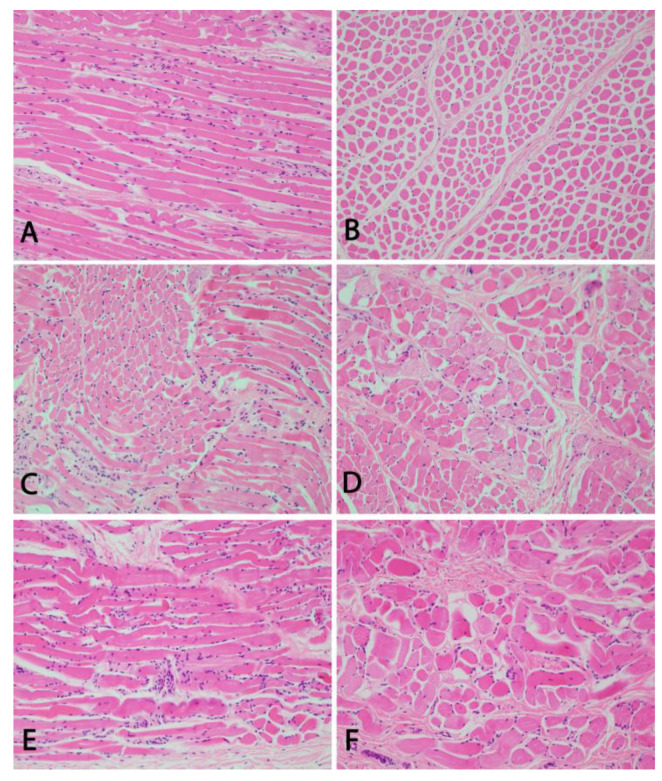
Histopathological features of BCSE-affected Holstein cattle. Longitudinal (**A**) and cross (**B**) sections of muscle fibers of the *M. retractor bulb.* The cross section (**B**) shows mild variation in size and cellular infiltrates. Longitudinal (**C**) and cross (**D**) sections of muscle fibers of the *M. rectus lateralis* show a moderate degeneration and atrophy with size variation of fibers. Severe degeneration with internalization of satellite cells and prominent variation in fiber size are seen in the longitudinal (**E**) and cross (**F**) sections of muscle fibers of the *M. rectus dorsalis*. 200× magnification, HE stain.

Two of the affected animals (Figure 1) used in our study were available for histopathological and one of these for neuropathological examination. As prior studies reported pathological changes in the *N. abducens* and the *Mm. recti lateralis et medialis*, all seven ocular muscles as well as the ocular globes of two affected animals were examined histologically. Gross examination and microscopic examination with hematoxylin and eosin stain (HE) were conducted. After longitudinal and cross section of muscle fibers, pictures were taken of the *M. retractor bulbi*, the *M. rectus lateralis* and the *M. rectus dorsalis* (Figure 2). Furthermore, the *N. opticus*, the *N. oculomotorius*, the *Chiasma opticum*, the *Tractus opticus*, the *Mesencephalon*, the *Medulla oblongata* including the *Nuclei vestibulares* and the *Nucleus nervi oculomotorii*, the *Thalamus* including the *Corpus geniculatum laterale* and the *Lobus occipitalis* were examined neuropathologically in one animal.

### 2.3. SNP Genotyping, Imputation and Genome-Wide Association Study (GWAS)

All animals were genotyped using the Illumina BovineHD BeadChip comprising a total of 777,962 markers. Quality control was accomplished using Plink 1.9 [13]. Only autosomal markers with a known position according to genome build UMD3.1 were kept and filtered using thresholds of 2% missing genotypes per marker and a minor allele frequency of 0.05. This resulted in a total of 543,241 SNPs remaining for further analyses. Based on these genotypes, all animals were imputed to sequence level, using the Run 5 of the database of the 1000 Bull Genomes consortium as a reference panel, comprising data for 1682 animals of around 70 different breeds and over 60 million variants [14] Imputation to full genome sequence was accomplished using the software Beagle 5.0 with default parameters [15]. After filtering for minor allele frequency with a threshold of 0.05, 10,772,372 imputed variants remained for further analyses.

A genome-wide single marker association analysis was conducted as a logistic regression as implemented in Plink 1.9 [13]. To correct for possible stratification, a multidimensional scaling, as also implemented in Plink 1.9 [13], was conducted using a genomic relationship matrix based on the high-density SNP array data and the first four axes of variation were included as covariates in the model. The number of dimensions to include was determined by visual inspection of a scree plot. The genome-wide significance threshold was determined by Bonferroni correction (*p* ≤ 0.05/number of markers). Visualization was carried out with R [16,17]. Significantly associated sequence variants were subjected to variant effect prediction using the VEP pipeline provided by Ensembl (https://www.ensembl.org/info/docs/tools/vep/index.html, accessed on 1 October 2020) based on annotation release 94 for the bovine UMD3.1 genome assembly (http://oct2018.archive.ensembl.org/Bos_taurus/Info/Index, accessed on 1 October 2020).

### 2.4. Evaluation of the Molecular Consequences of Amino Acid Substitutions

Sorting Intolerant From Tolerant (SIFT) [18] was used to predict the biological consequences of variants on protein level, whereby the SIFT score is a normalized probability of observing the new amino acid at a particular position estimated based on the comparison of homologous protein sequences [18].

## 3. Results

### 3.1. Clinical Phenotype

Out of 73 German and Swiss Holstein cows reported with eye anomalies resembling BCSE, 52 unambiguously affected animals (Figure 1) were selected as cases for the genetic analysis. Twenty-one BCSE-suspicious animals were excluded because the assessment of the medical condition by means of photographs and recorded case history showed that they did not fit the three underlying criteria of bilateral manifestation, simultaneous occurrence of convergent strabismus and exophthalmos.

### 3.2. Pathological Phenotype

Gross examination of the head of the two examined animals revealed a ventromedial strabismus. Ocular muscles and globes were unremarkable on gross examination. All seven eye muscles of the two examined cases showed similar histological changes of moderate to severe degeneration and regeneration (Figure 2). Multifocally, myocytes were hypereosinophilic with loss of cross striation and on cross section, groups of fibers appeared smaller in size and angular, which is compatible with atrophy of these fibers. Some myocytes showed pale eosinophilic change of the sarcoplasm (hyaline degeneration) and few degenerated myocytes were surrounded by macrophages. There were optically empty spaces separating the fibers and bundles of fibers. Rowing and internalization of nuclei indicate scarce regeneration of myocytes. Multifocally, perivascular infiltrates composed of lymphocytes, macrophages and fewer plasma cells and sparse neutrophilic and eosinophilic granulocytes. The left *M. retractor bulbi* of one animal did show only mild signs of degeneration and cellular infiltration. The neuropathological examination of one BCSE-affected animal did not reveal any morphological changes.

### 3.3. Genome-Wide Association Study and Candiate Genes

**Figure 3:**
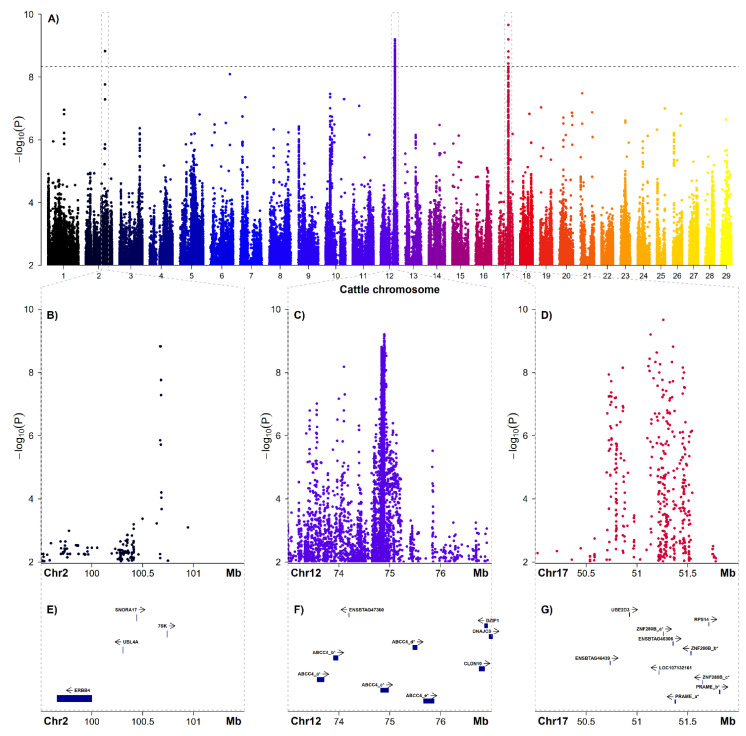
Results of the GWAS for BCSE in Holstein cattle based on imputed sequence level genotypes. (**A**) Genome-wide Manhattan plot of the GWAS results depicting the negative decadic logarithms of the *p*-values with respect to genomic position (UMD3.1). The horizontal dashed line represents the genome-wide significance threshold according to Bonferroni correction (pBonf < 0.05). Zoom into the three genome-wide significantly associated genome regions on chromosomes (Chr) 2 (**B**), 12 (**C**) and 17 (**D**). Gene content of the three BCSE-associated genome regions on Chr 2 (**E**), 12 (**F**) and 17 (**G**) according to Ensembl release 94 (UMD3.1, https://oct2018.archive.ensembl.org/Bos_taurus/Info/Index, accessed on 1 October 2020). Where no cattle gene symbols were available, human orthologue information was used or otherwise only the Ensembl ID is given. Note that genes marked with an asterisk and a subsequent letter do represent multiple genes annotated in the cattle genome with the same human orthologue. For further details, see Supplementary Material 2.

Initially, a GWAS including 52 affected animals and 95 controls was carried out using the pruned Illumina BovineHD BeadChip data comprising 543,241 autosomal SNP markers, but no genome-wide significant association signals were detected. In a second step, a GWAS was carried out based on imputed sequence level genotypes considering a total of 10,772,372 imputed SNP and InDel markers after filtering (Figure 3 and Table 1). This led to the discovery of significantly associated SNPs located at three different genome regions on chromosomes 2, 12 and 17 (Figure 3A). In total, 222 SNPs were above the genome-wide significance level (Table 1 and Supplementary Material 1).

On chromosome 2, six SNPs were above the genome-wide significance level, located within a stretch of 4591 base pairs (bp) ranging from 100,672,388 to 100,676,979 bp. On chromosome 12, a total of 211 SNPs were surpassing the genome-wide significance threshold, located between 74,836,987 and 74,930,904 bp. On chromosome 17, five genome-wide significantly associated SNPs were detected between 51,125,772 and 51,356,292 bp (Figure 3).

None of the significantly associated variants at 100.7 Mb on chromosome 2 are located within a gene (Figure 3E). Most of the 211 best-associated variants on chromosome 12 at 74.9 Mb are located within introns of the *ABCC4* gene (ATP-binding cassette subfamily C member 4, ENSBTAG00000023309) (Figure 3F, Table 1 and Supplementary Material 1). Among the other significantly associated markers in the region of the *ABCC4* gene are also coding variants such as a splice region variant (rs379903332), a synonymous variant (rs378129168) and two missense variants, namely p.Thr281Ile (rs385235934) and p.Ala360Thr (rs378531064). Notably, the p.Thr281Ile exchange affects a conserved residue and is predicted to be deleterious with a SIFT score of zero. In addition, one of the significantly associated variants located on chromosome 17 (rs377905476) is a splice region variant within the unknown gene ENSBTAG00000046306.

## 4. Discussion

Sporadic cases of anomalies in cattle that are not visible at birth but manifest later in life are mostly not reported or if so, not further investigated in detail. Nonetheless, affected animals might be impaired in production or health. In this study, we performed a comprehensive clinical, pathological and genetic investigation of Holstein cows from Germany and Switzerland showing BCSE, a possibly inherited eye disorder that was previously described in German Braunvieh cattle. It is postulated that this bovine condition resembles human progressive external ophthalmoplegia (PEO) representing a rare disease of heterogeneous origin.

In this study, the BCSE condition in Holstein cows was characterized clinically as well as histo- and neuropathologically. The obtained results confirmed the findings that were presented before in BCSE-affected German Braunvieh cattle. Histopathological examination of the ocular muscles of two BCSE-affected Holstein cows did reveal signs of degeneration and cellular infiltration. This could resemble human ocular myositis, which is described as an idiopathic inflammation of the extraocular muscles and can be a cause for strabismus [19]. Previous studies have, however, shown the presence of RRFs in ocular muscles of affected animals [7]. The appearance of these fibers is due to the accumulation of abnormal mitochondria, which can result from mitochondrial defects, but also from other conditions impairing muscle metabolism. As previously postulated, this resembles inherited forms of human progressive external ophthalmoplegia (PEO) [11,20]. We were, however, not able to verify the presence of RRFs in our samples as it was not possible to produce the required Gomori trichrome stained cryosections from our material.

The findings in conjunction with the angular appearance of the defect could also support the hypothesis that muscular atrophy and the resulting motoric insufficiency might be associated with neurodegenerative processes. The neuropathological examination did not, however, reveal morphological changes.

A total of 52 clinically affected animals, collected in two different countries, were used as cases in the GWAS. While 21 of these were directly diagnosed with BCSE by one of the investigators, the assessment of the remaining animals was only possible based on owner records and photographs. In order to include only unambiguously affected cases, the selection took place based on three stringent criteria including bilateral strabismus, simultaneous convergent strabismus and exophthalmos and first appearance at age > 6 months. This resulted in an additional 31 animals being included and 21 animals were considered as BCSE-affected further. This strict procedure was necessary, because several other possible causes of strabismus or exophthalmos are discussed [9]. Abnormalities in globe position in cattle can be found bi- or unilaterally and as convergent or divergent strabismus. Bilateral divergent strabismus, for example, may occur in association with hydrocephalus. Bilateral dorsomedial strabismus is suggestive of polioencephalomalacia [21]. Unilateral strabismus can be caused by tumors, inflammation or traumata. A total of 95 unaffected animals were selected as control for GWAS from our data repository. There were no anomalies of the eyes reported for those animals and they were selected to be more than two years old to exclude animals with mild initial signs of BCSE. As deep pedigree data of the cases were only partly available, we were not able to conduct a detailed pedigree analysis. Thus, we cannot conclude on a possible mode of inheritance as it has been described for Braunvieh cattle [8].

The initial GWAS using 543,241 markers from a high-density SNP array did not reveal any significant association signal. Subsequently, after extension of the GWAS data by imputation to sequence level revealing genotypes for more than 10.8 million SNPs and InDels, three genome-wide significant association signals located on different chromosomes were detected. The associated regions on chromosomes 2, 12 and 17 are not in line with previous mapping results for BCSE performed in German Braunvieh cattle [5,10]. In that population, positional cloning showed linkage to other genomic regions on two different bovine chromosomes indicating an independent genetic origin of BCSE in the Holstein breed.

Although linkage disequilibrium (LD) can be considered high in the global Holstein population [22], marker density of the used high-density array was apparently too low to obtain significant association for BCSE. Given the number of ~550 k informative SNPs included in the study, the average inter-marker distance is approximately 5 kb. Interestingly, the finally obtained association signal on chromosome 2 falls into a 60 kb gap in array-marker coverage. The same holds true for the GWAS hit on chromosome 12 as there was only one marker located between 74 and 75 Mb in the initial dataset. In addition, the association signal on chromosome 17 is located in a coverage gap of roughly 500 kb without any marker of the high-density array. The size of the gap on chromosome 2 is in a range similar to inter-marker distances on medium density SNP-arrays, which might indicate that haplotype blocks containing causative variants are rather small and represent historically distant events of mutation [22,23,24]. The other gaps are rather large, which prevent detection of the association signals. Given that the accuracy of imputation in the scenario implemented here can be considered high [25], the chosen approach nicely demonstrates the usefulness of imputed sequence level genotypes in mapping studies.

Based on our own observation that affected animals can occasionally be observed in many, if not most, Holstein herds, we assume that the defect allele frequency is comparatively high. Thus, although we cannot exclude the possibility that our GWAS detects variants only tagging the causative haplotype, we assume that the variant segregates in the reference panel and is imputed into the cases.

The final GWAS results indicate possible genetic heterogeneity causing BCSE in Holstein cattle. As a result that all significantly associated variants located on chromosome 2 are intergenic, and those on chromosome 17 are located within a protein coding gene of unknown function (ENSBTAG00000046306), we speculate that the association signal on chromosome 12 located in the region of the bovine *ABCC4* gene might be of special interest. The *ABCC4* gene codes for the multi drug resistance protein 4 (MRP4), which acts as an export pump that contributes to cellular detoxification [26] and thus has important implications in drug pharmacokinetics [27]. However, a functional link with the development of BCSE remains inconclusive. The encoded MRP4 protein belongs to a large family of transmembrane proteins, the C subfamily of ABC transporters, involved in active transport of substrates out of cells by functioning as an efflux pump. MRP4 transports a wide variety of compounds out of cells, some of which usually induce oxidative stress [28]. Despite the fact that *Mrp4*-deficient mice are born viable and do not show any obvious anomalies, it is known that they express abnormal reactions to various types of stress including retinal dystrophy [29]. Therefore, it was recently stated that MRP4 may play a role in the process of aging [30]. In light of the obtained GWAS results, one could therefore speculate that variants in the bovine *ABCC4* might have an impact on the development of BCSE in cattle, an anomaly that continuously progresses during aging. As the animals are, however, still comparatively young at the age of onset, this remains speculative.

The fact that the GWAS was conducted using imputed sequence level genotypes has, however, to be taken into account. Although the disease appears to be quite common in Holstein cattle, it seems unlikely that the putative causative variant(s) is/are contained in the reference panel and cannot be imputed. Thus, we screened the vicinity of the GWAS signals for other functionally plausible candidate genes.

On chromosome 17, the *nuclear receptor corepressor 2* (*NCOR2*) gene is located about 2 Mb downstream of the association signal. *NCOR2* is a corepressor, which influences multiple physiological pathways. It is a silencing-mediator for retinoid and thyroid hormone receptors, therefore having the alternative name Silencing Mediator for Retinoid and Thyroid Hormone Receptors (SMRT). *NCOR2* has a wide-ranging role in developmental as well as homeostatic processes [31,32]. For example, an analysis of *SMRT* knock-out mice revealed that SMRT plays a critical role in forebrain development and also in maintenance of the neural stem cell state [33]. SMRT represses the expression of the *JMJD3* gene that functions as a histone H3 trimethyl K27 demethylase which acts as a critical activator of neurogenesis from adult subventricular zone neural stem cells (NSCs) and therefore takes part in the epigenetic mechanisms that enable lifelong neurogenesis from (NSCs) [33,34]. Furthermore, Ataxin 1 (ATX1), a polyglutamine protein whose mutant form causes type 1 spinocerebellar ataxia (SCA1) in humans (OMIM 164400), was found to be functionally linked to SMRT [35]. SCA1 is a progressive neurodegenerative disease in humans pathologically characterized by ataxia, progressive motor deterioration and loss of Purkinje cells [35,36]. Those results indicate that SMRT has many biological properties that in some cases can be associated with neurodegenerative conditions. In order to establish a potential functional link with BCSE, however, the hypothesis that the disease is of neurodegenerative origin and not due to myositis needs to be further tested.

Finally, approximately 2 Mb downstream of the association signal on chromosome 12, the DnaJ heat shock protein family (Hsp40) member C3 (*DNAJC3*) gene is located. This gene has been mainly implicated in the development of diabetes [37,38], but also neurodegeneration [37]. The pathomechanism of diabetes caused by *DNAJC3* mutations further involves mitochondrial degeneration [39]. Thus, it could be also speculated that this gene might be functionally linked to the development of BCSE in cattle.

## 5. Conclusions

For the first time, a cohort of BCSE-affected cows in the Holstein breed is presented. The clinicopathological phenotype is highly similar to BCSE described in German Braunvieh cattle and demonstrated degeneration and cellular infiltration in the eye muscles. Only by using imputed sequence level genotype data, genome-wide significant GWAS hits were revealed on three different chromosomes highlighting the usefulness of this approach for mapping studies. The associated genome regions include the *ABCC4* gene as well as markers adjacent to the *NCOR2* and *DNAJC3* genes. Our results challenge the claim of a monogenic mode of inheritance and suggest a more complex inheritance of BCSE in Holstein cattle. Furthermore, in comparison to previous results from German Braunvieh cattle, it illustrates an obvious genetic heterogeneity causing BSCE in cattle.

## Figures and Tables

**Table 1:** Top 20 of the genome-wide significantly associated variants (P_Bonf_ ≤ 0.05). Bold lines indicate associations with P_Bonf_ ≤ 0.01.

Chr	rsID	Position [bp] ^1^	Alleles ^2^	MAF ^3^	Odds Ratio	*p*-Value ^4^	VEP ^5^	Gene
2	rs136316260	100,672,388	G/T	0.17	38.75	1.49E-09	intergenic	
2	rs134704382	100,674,312	G/A	0.17	38.75	1.49E-09	intergenic	
2	rs135186290	100,675,331	A/G	0.17	38.75	1.49E-09	intergenic	
2	rs134623922	100,675,365	G/A	0.17	38.75	1.49E-09	intergenic	
2	rs385880764	100,675,837	-/ATC	0.17	38.75	1.49E-09	intergenic	
2	rs133964128	100,676,979	A/G	0.17	38.75	1.49E-09	intergenic	
12	rs377992474	74,888,457	T/A	0.34	9.90	8.36E-10	intron	*ABCC4*
12	rs468443940	74,891,825	T/G	0.36	8.76	1.28E-09	intron	*ABCC4*
12	rs381451112	74,892,681	C/G	0.34	10.78	1.41E-09	intron	*ABCC4*
12	rs383448105	74,892,723	A/G	0.34	10.04	6.27E-10	intron	*ABCC4*
12	rs378170580	74,892,729	T/G	0.34	10.84	1.00E-09	intron	*ABCC4*
12	rs384157620	74,892,843	A/G	0.34	9.68	1.09E-09	intron	*ABCC4*
12	rs110869430	74,893,319	T/G	0.33	9.70	7.05E-10	intron	*ABCC4*
12	rs109771712	74,893,351	C/G	0.32	9.64	9.94E-10	intron	*ABCC4*
12	rs876085957	74,893,362	C/T	0.34	9.86	1.09E-09	intron	*ABCC4*
12	rs110191959	74,894,971	A/G	0.31	12.26	1.46E-09	intron	*ABCC4*
12	rs384153179	74,895,060	T/C	0.32	11.94	1.55E-09	intron	*ABCC4*
17	rs451202354	51,135,057	C/T	0.17	153.30	6.29E-10	intergenic	
17	rs384954012	51,259,815	A/-	0.17	90.24	2.17E-10	intergenic	
17	rs377905476	51,356,292	G/T	0.18	45.64	1.53E-09	splice region	*ENSBTAG00000046306*

^1^ Genome assembly UMD3.1; ^2^ minor allele given first; ^3^ minor allele frequency; ^4^ raw *p*-value; ^5^ variant effect prediction (VEP). For further details, see Supplementary Materials 1 and 2.

## Data Availability

The raw data are available upon reasonable request from the corresponding author. Genotype data obtained from the 1000 Bull Genomes Consortium as reference for imputation are not publicly available.

## Supplementary Materials

The following are available online at https://www.mdpi.com/article/10.3390/genes12071039/s1, Supplementary Material 1: Variants genome-wide significantly associated (P_Bonf_ ≤ 0.05) with BCSE. For each variant, rsID, position, association results and variant effect prediction are given. Bold faced lines indicate variants with P_Bonf_ ≤ 0.01. Supplementary Material 2: Genes located in the associated genome regions on BTA2, 12 and 17 (see also Figure 3).
